# Mini-Review of Laboratory Operations in Biobanking: Building Biobanking Resources for Translational Research

**DOI:** 10.3389/fpubh.2020.00362

**Published:** 2020-07-28

**Authors:** Mine S. Cicek, Janet E. Olson

**Affiliations:** ^1^Department of Laboratory Medicine and Pathology, Mayo Clinic, Rochester, MN, United States; ^2^Department of Health Sciences Research, Mayo Clinic, Rochester, MN, United States

**Keywords:** disaster and risk management, biobanking and biorepositories, laboratory information management system (LIMS), biorepository operations, biospecimen research

## Abstract

Biobanks have become integral to improving population health. We are in a new era in medicine as patients, health professionals, and researchers increasingly collaborate to gain new knowledge and explore new paradigms for diagnosing and treating disease. Many large-scale biobanking efforts are underway worldwide at the institutional, national, and even international level. When linked with subject data from questionnaires and medical records, biobanks serve as valuable resources in translational research. A biobank must have high quality samples that meet researcher's needs. Biobank laboratory operations require an enormous amount of support—from lab and storage space, information technology expertise, and a laboratory management information system to logistics for sample movement, quality management systems, and appropriate facilities. A paramount metric of success for a biobank is the concept of every biospecimen coming to the repository belongs to a participant who has something to contribute to research for a healthier future. This article will discuss the importance of biorepository operations, specific to the collection and storage of participants materials. Specific focus will be given to maintaining the quality of samples, along with the various levels of support biorepositories need to fulfill their purpose and ensure the integrity of each specimen is maintained.

## Introduction

**Figure 1:**
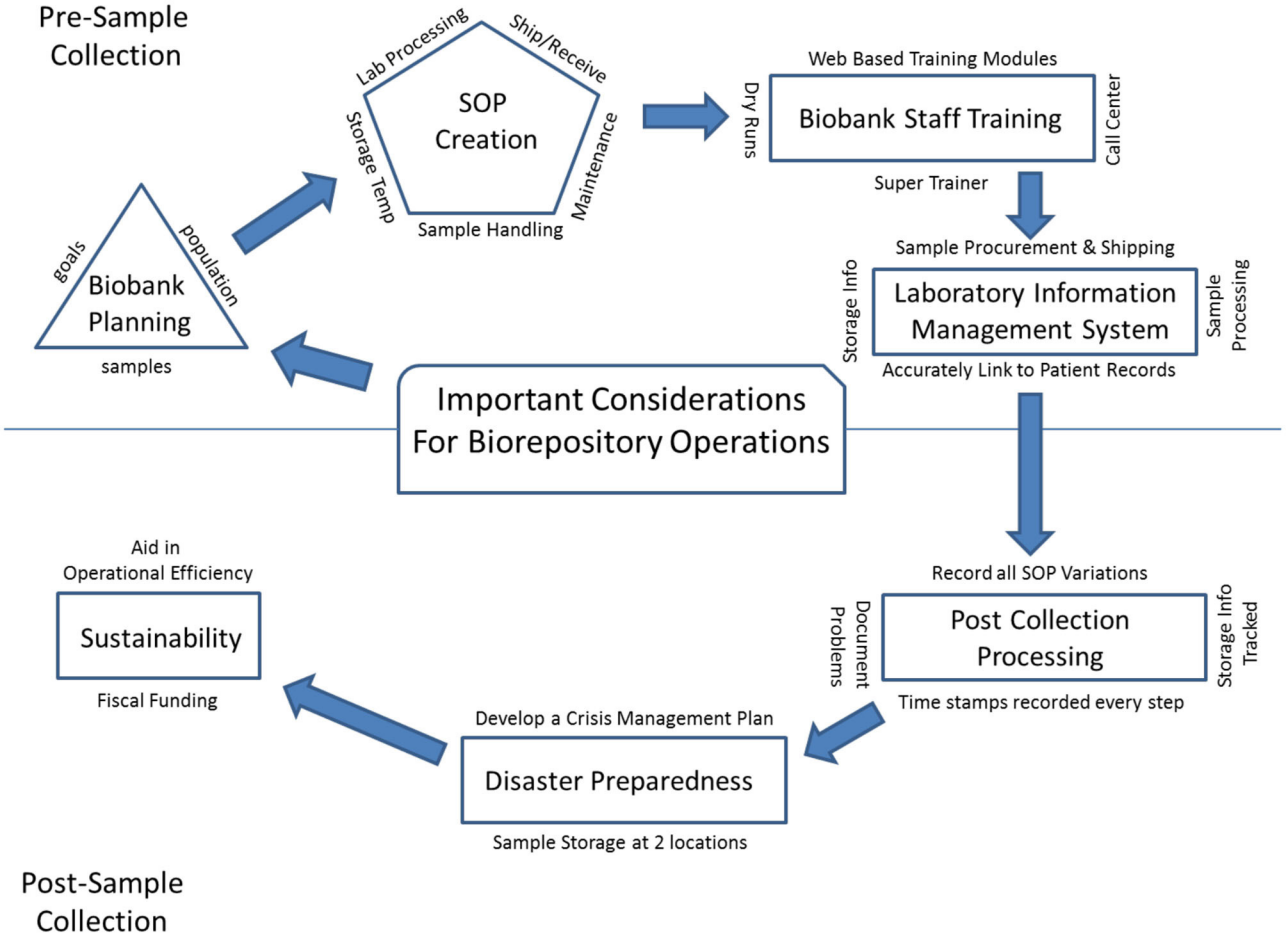
An overview of important considerations for biorepository operations.

In the past few decades as technology and informatics has expanded, scientific research has moved from laboratory-based discovery to translational research, seeking to identify underlying biologic causes for disease and offer personalized treatment for patients. Large numbers of samples, procured from study populations that can be followed across time, have become the critical component for translational research, and the twenty-first century has shown the emergence of biobanks around the world. There are now both large population-based biobanks, and patient-based biobanks including the Kadoorie Biobank in China (1), LifeGene in Sweden (2), CONOR and MoBa in Norway (3, 4), Auria in Finland (5), UK Biobank (6), Estonian Biobank (7), BioBank Japan (8), Korean Biobank (9, 10), Taiwan Biobank (11), and Million Veteran Program (12) and All of Us Biobank (13) in the United States. Biobanks must collect, process, store, and disseminate biologic material with appropriate and complete annotation in order to meet the needs of future translational researchers. A successful translational research program depends on the existence of biobanks with solid infrastructure that can provide the needed consistency in specimen collection, specimen tracking, quality control (QC) management, sample storage, as well as disaster recovery plans and long-term financial stability. These key elements are building blocks for any biobank to be successful and for translational research to thrive. Guidelines have been established for best practices in biobanking and for biospecimen resource creation by both NCI (14) and ISBER (15). Here we provide an overview of important considerations for biorepository operations (Figure 1), defined herein as operational groups that support biospecimen collection and storage across multiple biobanks.

## Biobank Planning

Prior to initiation of a new biobank collection, the biorepository operational team must obtain an understanding of the new biobank's goals, subject population(s), collection and processing protocols, storage requirements, pre-analytical data collection need and long term monitoring requirements. The biobank should have a clear plan to ensure that it can successfully meet its own collection goals as well as anticipated needs of both current and future translational researchers. The biorepository operation team's role begins early on at the protocol design stage before the first participant volunteers to take part in the biobank. Operational procedures must be established and tested to ensure each specimen will be collected and processed properly and in a timely manner to guarantee sample integrity. Sample integrity refers to the consistent processing of a specimen to ensure all collected materials remain intact and usable for the purpose for which they were collected. This integrity is tracked by data that is captured at each step of the collection and storage process. It allows for a history of the sample to be recorded to ensure data that will eventually be collected by researchers resulting in both meaningful and consistent results. That nothing happened in sample processing or storage that might alter these results. The burden of responsibility resides equally on the staff of the biorepository and biobank (study) to conduct the collection site feasibility assessment, establish of standard operating procedures (SOP) and train personnel involved in sample procurement.

## SOP Creation

SOPs should be developed to ensure high quality of the specimen collected and consistent sample handling to reduce pre-analytical variables, including pre-processing time, shipping temperature, centrifugation speed and temperature, processing time and storage temperature. Some SOPs fall to the biobank collection team, but many fall to the biorepository staff to implement, including biospecimen handling, laboratory processing, shipping and receiving methodology, documentation within a record management system, equipment maintenance support and facility security (16). Moore et al. reported in 2011 that many factors in biospecimen handling can affect downstream applications (17). These handling effects can influence translational research results, thus Moore et al. recommended that all published results from experiments should also include a report on how samples were handled during collection, otherwise known as BRISQ (Biospecimen Reporting for Improved Study Quality).

The biorepository operations team should assist the biobank team to evaluate collection tube options, and test if necessary, to determine the appropriate collection protocol which will allow the specimens collected to give the optimal desired final product for the goal of the biobank. Processing instructions and allowable time post collection should be defined clearly in SOPs based on literature and manufacturer's recommendations. In order to maintain molecular stability, samples should be stabilized or processed as soon as possible after collection (18). However, different collected biospecimens will inherently have different processing requirements. Elliott et al. for example, describes testing done prior to the inception of the collection of UK Biobank samples (6). One test examined the length of time samples could be stored at 4°C before detectable differences in important analytes arose. Results demonstrated that a time window of 24 h showed no differences, but at the time mark of 36 h, differences were present. Thus, the final SOP was written to reflect those findings stating that the desired time to cryopreservation of aliquots should be no more than 24 h after collection (6). Once established, all SOPs should be reviewed annually and updated as needed.

## Biobank Staff Training

Sample integrity can be maximized by adherence to established SOPs as described above, along with properly training its staff. Staff training and periodic review of the overall process is especially important for biobanks that rely on multiple accrual sites for initial patient contact, specimen procurement, and pre-shipment processing, where variations in procedures could happen based on site infrastructure. Training of biobank accrual site staff should include a super-trainer who would serve to train new staff, in anticipation of staff turnover throughout the project. Web based training modules can also be helpful to biobank accrual sites, to provide continuing support and information regarding SOP updates. The remote ongoing support can be established via call center, where questions are triaged to the appropriate personnel to be answered in a timely manner. Finally, dry runs can be executed as part of onboarding new collection sites, to ensure the process flowed smoothly through shipment prior to collection. This model has resulted in very successful accrual of quality samples in other biobanks.

## Laboratory Information Management Systems

Biorepository operations daily activity depends on a reliable sample tracking, laboratory information management system (LIMS). Having a well-established information technology (IT) LIMS in place is critical to ensure sample data integrity similar to sample quality. The end to end life cycle of a biobank collection lives in a LIMS system where each collection sample is linked to the correct patient records, from the time of procurement, through shipment, processing, to final long-term storage (18). Indeed, for large biobanks, there is an absolute requirement for an IT LIMS for specimen tracking. Methodology and use of an integrated IT LIMS, along with mode of information entry, can be accomplished in a variety of ways. For example, the Estonian Biobank reports having a recruiter fill out the questionnaire with the participant at time of donation, creating a record (7). When required fields were not filled in properly, the recruiter was alerted in real time allowing immediate correction. This is one way to ensure all information is both complete and accurate. The UK Biobank, a different method, assigns a unique bar code to each sample and scans it in at the assessment center into their IT system, to link it with the participant's unique ID which was assigned during enrollment. In this approach the bar-coded tubes are not preassigned to a subject to avoid participant identification errors and prevent empty blood collection tubes being logged in. Scanned in bar codes at the time of collection also automatically initiate a timer built into the IT system of the UK biobank, so time can be automatically, instead of manually, linked (6). Biobank Japan has independent medical coordinators who collect the information and then anonymize the specimens at the collection site (8). Thus, IT systems and how they are used can be different across biobanks, but are always crucial to ensure accurate record keeping during sample procurement.

## Post-Collection Processing

After biospecimens are collected, protocols may require some biospecimens to undergo minimal processing to ensure the stability of the specimen; other biospecimens may be stored at a specified temperature before being shipped to a processing center or to the main biobank site. Any processing at collection sites should be documented in the IT system, including time of processing and any problems or variations from the published SOP. Methods and timing for shipment from accrual sites to the biobanks also vary based on the sample type stability. Sample tracking through the IT system remains critical, so sample arrival time along with temperature upon delivery, should all be recorded for each specimen. For example, samples in the Kadoorie Biobank, are placed at 4°C for a few hours until processing, then are stored at −40°C for 3–4 months, before being couriered on dry ice to a central blood repository for long term storage at −80°C (1). The Million Veteran Program ships whole blood to a processing center for same day DNA extraction (12). Samples collected for the UK Biobank have minimal processing done at collection sites and are stabilized at the appropriate temperatures, before being sent day of collection to high throughput sites for completion of processing (6).

Biobanks often use a commercial courier for overnight specimen shipments. Commercial couriers are the preferred method of transportation for specimens as they are trained with their own SOPs in temperature control and expedited specimen delivery. Upon arrival at the Biobank, each sample package must go through a quality check for temperature integrity and package damage. The samples received must be reconciled with the attached requisition form. All deviations observed should be recorded in the LIMS against samples.

The quality of a biorepository operations should also be measured by laboratory infrastructure. Large biobanks will have to invest on instrumentation to minimize human errors and automate sample processing and storage (12, 19). Robotics, if appropriate, can provide varying advantages such as reducing cost, improving reproducibility, and providing a better overall quality assurance. Robotics also can be helpful in sample annotation and tracking in LIMS. Robotic retrieval of samples can ensure temperature stability.

The goal of biorepository operations management is to maintain the highest quality specimen available for translational research. Protocol deviations result in preanalytical variability that may impact downstream use of samples (20). In 2000, Narayanan published a detailed review of three areas of pre-analytic variability: physiologic, specimen collection, and influence of interference factors that need to be considered during specimen collection (21). Clearly some of the burden of minimizing pre-analytic variables will fall upon the biorepository. Numerous articles and reviews have been published reviewing and addressing best practices for biobanks specifically in areas of sample collection and processing, tracking, and storage (18, 21–23) to help guide biobanks and are available as resources. Quality management can also be maintained through the use of routine testing and QC metrics of the processed specimens, as well as the laboratory robotics. Any variance from SOPs as well as regular QC and maintenance records should be documented. Finally, the biorepository staff should conduct periodic review of the biobanking literature to ensure that SOPs are updated with the latest advancements based on newly published reports and new technology available.

Even with all of this standardization, each biorepository operational teams will vary somewhat in their methods. However, specimens from different biobanks must have common requirements and metrics to ensure the quality of all specimens at all biobanks, especially when procedures may vary. The Biorepository Accreditation Program (BAP), created by CAP, serves to provide requirements for standardization of biospecimen processes that will result in high quality specimens (24). Accreditation is a 2 years cyclical process, involving 1 year of on-site inspections by certified peers, who review documents, observe practices, and ask questions of staff, and then 1 year of self-reported inspections. Checklists containing standards are evaluated and though exact protocols may vary from lab to lab, all must be complete. Specific personnel qualifications, training and competency assessments also must be met (24). Checklists and findings are reviewed and recommendations are given to fix deficiencies found. As of 2018, 53 biorepositories are fully CAP BAP accredited with many more in the review phase.

## Disaster Preparedness

Biobanks represent an irreplaceable asset, and consideration should be given toward preparation for possible urgent circumstances such as fires, floods, tornados or hurricanes (25–28). The COVID-19 pandemic of 2019–2020 provided a unique challenge of a different sort. Instead of reacting to a natural disaster that may involve structural damages, or power outages, this pandemic affects sample collection, and sample processing. Decisions needed to be made to assess if sample collections could still continue. Would at home collections and shipments still be possible especially to ensure sample integrity? Planning is essential to protect the time and value of yet to be collected specimens volunteered as well. ISBER recommends that biobanks have an emergency management plan and a disaster contingency plan (29). The establishment of a crisis management team is needed to identify and assess risks specific to each Biobank and to create a risk response as part of an established contingency plan, should an identified risk occur (29). For example, could virtual data collection be done during a pandemic? How could sample integrity be ensured if at home specimen collection was done? How shipments could be ensured. For a more in-depth review, see Parry-Jones (29), which describes how a crisis management plan can be developed. Along with this plan, storing biologic samples along with electronic records at two geographically separated locations is another way to secure samples and ensure a biobank can withstand a disaster without losing the complete collection.

## Sustainability

Finally, long term biobank sustainability must be considered. The NCI Biorepositories and Biospecimen Research Branch developed a web-based application (the Biobank Economic Modeling Tool) to enable determination of the accurate cost recovery fee to help with long range planning (30). To keep the Biobank running long term, it is essential that biobanks accurately assess financial costs for distribution of samples. Also, planning to aid in the operational efficiency of a biobank is critical. According to Watson et al. (31) this includes input efficiency (patient enrollment and biospecimen accrual), internal efficiency (optimizing processing, balancing resources to support retrospective questions while not storing beyond what is anticipated to be useful), and offering more data elements (like records of pre-analytic variables) The more samples that are requested from a biobank, the more financial compensation it can receive. Securing the social adaptability of the Biobank will allow it to become a resource for all to use. Having an ethics review board that examines biobank and research projects, along with a commitment to good practices, and openness with the public about usage and returned results (if appropriate) is critical.

## Summary

Biobanks have become integral to improving population health. We are in a new era in medicine as patients, health professionals, and researchers increasingly collaborate to gain new knowledge and explore new paradigms for diagnosing and treating disease. Many large-scale biobanking efforts are underway worldwide at the institutional, national, and even international level. When linked with subject data from questionnaires and medical records, biobanks serve as valuable resources in translational research. A biobank must have high quality samples that meet researcher's needs. Biorepository operations require an enormous amount of support—from lab and storage space, information technology expertise, and a laboratory management information system to logistics for sample movement, quality management systems, and appropriate facilities (Table 1). A paramount metric of success for a biobank is the concept of every biospecimen coming to the repository belongs to a participant who has something to contribute to research for a healthier future.

**Table 1:** Biorepository is a complex operation and requires multiple components to work in harmony to be successful.

**Components of a Biobank**	**Purpose**
Pre- planning process	To understand the goals of the biobank
Standard operating procedures (SOP)	To create protocol documents to ensure laboratory procedures are trained on and followed by all staff and to minimize pre-analytic variability in order to have quality specimens that meet the goals of the biobank
Biobank staff training	To ensure all staff involved in specimen collection, processing, and storage follow SOPs consistently
Lab information management system	To track a specimen from collection through processing, and storage and to link patient to sample records throughout the full life cycle of a sample
Disaster preparedness plan	To create a plan to ensure the biobank operations are ready to operate and keep samples safe under unexpected disasters
Sustainability	To create a plan to ensure that biobank operations is financially stable to be a long-term resource

## Author Contributions

MC: corresponding author, responsible for conception and design of the manuscript, drafted and edited, and responsible for answering questions and revisions. JO: equally accountable for editing and responsible for answering questions. All authors contributed to the article and approved the submitted version.

## Conflict of Interest

The authors declare that the research was conducted in the absence of any commercial or financial relationships that could be construed as a potential conflict of interest.
